# Speaking up about patient safety concerns: view of nursing students

**DOI:** 10.1186/s12913-022-08935-x

**Published:** 2022-12-19

**Authors:** Magdalena Hoffmann, Christine Maria Schwarz, David Schwappach, Chiara Banfi, Christoph Palli, Gerald Sendlhofer

**Affiliations:** 1 Executive Department for Quality and Risk Management, University Hospital Graz, Auenbruggerplatz 1, 8036 Graz, Austria; 2 Research Unit for Safety and Sustainability in Healthcare, c/o Division of Plastic, Aesthetic and Reconstructive Surgery, Department of Surgery, Medical University of Graz, Auenbruggerplatz 2, 8036 Graz, Austria; 3 Division of Endocrinology and Diabetology, Department of Internal Medicine, Medical University of Graz, Auenbruggerplatz 15, 8036 Graz, Austria; 4 Institute of Social and Preventive Medicine, University of Bern, Bern, Switzerland; 5 Institute for Medical Informatics, Statistics and Documentation, Medical University of Graz, Auenbruggerplatz 2, 8036 Graz, Austria; 6 Institute of Health Care and Nursing, University of Applied Sciences FH Joanneum, Alte Poststrasse 149, 8020 Graz, Austria

**Keywords:** Communication, Patient safety, Safety culture, Nursing students, Speak-up

## Abstract

**Background:**

“Speaking up” is considered an important patient safety behaviour. The main idea is to voice patient safety concerns; however, several studies revealed that the organisational culture can be obstructive. In previous studies, we already identified barriers for doctors, nurses and medical students. In the current study, we explore how nursing students use “speaking up” during their internship in an academic teaching hospital.

**Methods:**

Between 2019 and 2020, 212 nursing students were invited to take part in the survey. The validated Speaking Up about Patient Safety Questionnaire (SUPS-Q) was used to assess speaking up behaviours in nursing students. The SUPS-Q consisted of three behaviour related scales (11 items), three culture related scales (11 items), a question regarding barriers to speak up as well as a clinical vignette assessing a hypothetical speaking up situation.

**Results:**

In total, 118 nursing students took part in the survey (response rate: 56%). Most of them noticed specific safety concerns, observed errors or rule violations. The vignette was seen as very realistic and harmful to the patient. However, the majority responded that they did not speak up and remained silent. They reported a rather discouraging environment and high levels of resignation towards speaking up. However, more advanced students were less likely to speak up than less advanced students (*p* = 0.027). Most relevant barriers were fear of negative reaction (64%), reaction not predictable (62%) and ineffectiveness (42%).

**Conclusions:**

Survey results of nursing students imply that speaking-up behaviours and remaining silent are common behaviours and coexist in the same individual. The clinical vignette and barriers to speaking up revealed that a hierarchical system does not support speaking-up behaviours. Organizational development is needed to foster professional teamwork, support attentive listening, encourage critical thinking, and problem-solving skills.

**Supplementary Information:**

The online version contains supplementary material available at 10.1186/s12913-022-08935-x.

## Background

Medication errors, incorrect hand hygiene, surgery-related errors and many others can lead to serious patient harm [1–4]. Therefore, “speaking up” is considered an important patient safety behaviour for healthcare professionals and involves raising concerns verbally and in a timely manner [5]. The main purpose of speaking up is to be vocal about patient safety, however studies revealed that the organisational culture can be counterproductive [6, 7]. The ability to speak up depends on many factors and common barriers include i) the absence of audience, ii) power dynamics and authority gradients, iii) fears of damaging relationships as well as iv) feelings of resignation [6]. Organizations with psychological safety create the ability to learn, to be innovative and to thereby foster behaviours relevant to patient safety [7]. Speaking up can also positively influence inter-professional teamwork which influences the quality of care [8].

Among others, a prerequisite for increasing patient safety as well as the safety culture is education and continuous training in order to gain safety competencies [9]. In 2009, the World Health Organization (WHO) developed a patient safety curriculum guide for medical schools [10, 11]. Since then, several studies showed that integration of patient safety education into the undergraduate curriculum still displays potential for improvement [11, 12]. Nursing students self-reported a high importance of patient safety but they also reported that they do not display respective skills and knowledge to that level [12, 13]. A recent study found that patient safety education of nursing students was partially effective in improving long-term patient safety competencies [9].

Speaking-up behaviour is a determinant for patient safety culture of any health-care organization. Consequently, it is of high importance to investigate which barriers are present in a health-care organization across all disciplines. Understanding barriers might also support the implementation of measures to create a safer patient environment.

We performed two distinct surveys to gain knowledge of facilitators and barriers of speaking-up habits in an academic teaching hospital in Austria. Based on an established and validated survey instrument from the Swiss Patient Safety Foundation, behaviours and perceived speaking-up culture of doctors, nurses and medical students were assessed. In the first survey, doctors and nurses self-reported low levels of confidence for speaking up due to psychological pressure. Approximately half of them observed specific concerns, and up to 50% did not speak up in certain situations. The second survey among medical students found that more advanced students have higher concerns about patient safety than less advanced students. More than two thirds had specific safety concerns and noted that rules were neglected, as well as nearly half of them had observed at least an error. They also reported not having addressed critical situations due to certain barriers [10, 14].

Both surveys showed serious barriers for speaking-up and high psychological pressure in an academic teaching hospital. Due to a hierarchical systems and a dependency relationship of trainees in terms of training and assessment, speaking up seems difficult for all involved groups. A study in the United Kingdom by Rees et al. also showed that during an internship, nursing students were specifically asked to tolerate poor practice [15].

Due to the lack of data in Austria, the research question was to investigate nursing students’ speaking up behaviours and to compare it with previous studies at the same academic teaching hospital. Therefore, a survey among nursing students was performed with a well-known instrument. Differences in survey responses between assessed student groups (advanced versus less advanced) were analysed to understand whether attitudes and experiences related to speaking-up change over time and if progress and if experience is a relevant factor.

## Methods

### Reporting

The research and reporting methodology followed the Checklist for Reporting Of Survey Studies, recommended by the EQUATOR network.

### Study population

The study was conducted between 2019 and 2020. Data collection was performed between December 2019 and June 2020 over a period of 7 months (students have different attending times at University of Applied Sciences), using the validated “Speaking Up about Patient Safety Questionnaire” (SUPS-Q). Nursing students (*n* = 212) at the Department of Health Studies, Health Care and Nursing, University of Applied Sciences, Graz, Austria. In total, 212 nursing students were asked to participate in the survey.

Students of the study term 2017 were invited using an online-survey instrument (Evasys ©), whereas students of the study terms 2018 and 2019 were invited using a paper-based survey. The paper survey was handed out to all students by the administration of the Department of Health Studies and a box for survey collection was provided. Participation was on a voluntary basis. On the first page of the paper-based survey, an introduction about the aim of the study was given. By filling in the survey, participants agreed to take part in the study. Each potential participant was informed that collected data was going to be stored at the Department of Quality and Risk Management and that data analysis performed by the Medical University of Graz would be strictly anonymous.

### Survey instrument

The SUPS-Q is a validated questionnaire [6, 14, 16] that consists of three behaviour-related scales with 11 items each such as i) perceived safety concerns, ii) withholding voice and iii) past speaking-up behaviours. These items are scored on a five-point Likert scale from “never (0 times)” to “very often (> 10 times in the last four working weeks).

Speaking-up culture was assessed by 11 items using a seven-point Likert scale from “strongly disagree with this statement (value 1)” to “strongly agree with this statement (value 7)”. Higher scale scores indicate higher levels of perceived psychological safety, encouraging environment for speaking up and resignation towards speaking up.

### Data analysis

Furthermore, barriers for speaking up were assessed using six pre-defined reasons as well as a clinical vignette (missed hand disinfection by a senior physician) assessing a hypothetical speaking-up situation (seven-point Likert-scale).

### Data analysis

Categorical variables are displayed as absolute and relative frequencies, continuous variables with mean and standard deviation, unless stated otherwise. Scale scores were calculated as the mean of the scale items, including only participants that responded to more than half of the total amount of items in the scale. This strategy led to the exclusion of one participants from the calculation of the scale scores of “encouraging environment” and, and two participants from the “resignation toward speaking up”, respectively. The number of missing values is reported per item and per scale in Table 3.

Reliability was calculated by means of Cronbach’s alpha and Inter-item correlation. Following Schwappach et al. [14], the negatively worded items in the scale “resignation towards speaking up” were reverse-scored for calculating the total scale score and total scale reliability. Group differences between semester groups were computed with the Wilcoxon rank-sum test. A non-parametric test was chosen because it is suited for the comparison of ordinal data produced with Likert scales. Additionally, some item scores were not normally distributed. Results were compared to those obtained by independent sample t-tests. Both statistical approaches yielded comparable results.

A *p* value ≤ 0.05 was considered significant. All statistical analyses were conducted using R version 4.1.0 (https://www.r-project.org).

### Ethical considerations

The study was approved by the ethics committee of the Medical University of Graz (vote-number: 30-303ex 17/18).

## Results

Of the 212 invited nursing students, 118 took part in the survey (response rate = 56%). For four participants the information about the study semester was not available and was not considered for the comparison of semester groups. Thereof, 44 (39%) were attending the 2^nd^ or 3^rd^ and 70 (61%) the 5^th^ or 6^th^ semester. For further characteristics of the study sample, see Table 1.

**Table 1:** Characteristics of the study sample

**Total, n**		**118**
**Study term 2017, n (%)**		6 (5.1)
**Study term 2018, n (%)**		67 (56.8)
**Study term 2019, n (%)**		45 (38.1)
**Females, n (%)**		90 (78.3)
**Medical area of last internship, n (%)**	Surgery	20 (23.3)
	Pediatric	20 (23.3)
	Internal medicine	14 (16.3)
	Miscellaneous	15 (17.4)
	Neurology	12 (14.0)
	Gynaecology and obstetrics	5 (5.8)
	Missing values	32
**Semester attending, n (%)**	2^nd^	1 (0.9)
	3^rd^	43 (37.7)
	5^th^	64 (56.1)
	6^th^	6 (5.3)
	Missing values	4
**Semester attending, n (%)**	2^nd^ and 3^rd^	44 (38.6)
	5^th^ and 6^th^	70 (61.4)
	Missing values	4

### Perceived safety concerns, withholding voice and speaking up behaviours

In total, 94/117 (80%) of students had specific concerns, 84/117 (72%) observed an error that could have been harmful and 97/116 (84%) noticed rule violations during their four weeks period in the hospital. Furthermore, 83/117 (71%) decided to not bring up specific concerns, 86/117 (74%) kept ideas for improving patient safety for themselves and 82/117 (70%) did not address a colleague to follow specific patient safety rules. Of all students, 67/117 (57%) spoke up when they had information that might have prevented a safety incident in their unit. Concerning speaking up behaviour, 90/118 (76%) brought up specific concerns on patient safety, 86/118 (73%) addressed an error that could have been harmful, and 87/117 (74%) addressed a specific issue to colleagues. Finally, 63/116 (54%) believed that they prevented an incident by bringing up specific concerns, with a significantly higher tendency to do so among students attending the 2^nd^-3^rd^ vs. 5^th^-6^th^ semester (see Table 2).

**Table 2:** Absolute (n) and relative frequency (%) of perceived concerns, withholding voice and speaking up for the total group and stratified by study semesters

*In everyday work, it sometimes happens that things go wrong and risks to patients arise. This could be a result of medication error, poor hand hygiene or missing documentation. Over the last 4 weeks, how frequently..*							
	Groups	Never (%)	Rarely (%)	Sometimes (%)	Often (%)	Very often (%)	*p*
Perceived concerns (Cronbach ‘s alpha = 0.70, mean IIC = 0.46)							
…have you had specific concerns about patient safety?	Total	23 (20%)	56 (48%)	33 (28%)	4 (3%)	1 (1%)	
	Semester 5–6	15 (22%)	28 (41%)	23 (33%)	3 (4%)	0 (0%)	0.574^1^
	Semester 2–3	6 (14%)	28 (64%)	8 (18%)	1 (2%)	1 (2%)	
…have you observed an error which—if uncaptured—could be harmful to patients?	Total	33 (28%)	62 (53%)	20 (17%)	1 (1%)	1 (1%)	
	Semester 5–6	14 (20%)	44 (64%)	10 (14%)	1 (1%)	0 (0%)	0.244^1^
	Semester 2–3	18 (41%)	16 (36%)	9 (20%)	0 (0%)	1 (2%)	
…have you noticed that your workplace colleagues haven’t followed important patient safety rules, intentionally or unintentionally?	Total	19 (16%)	43 (37%)	32 (28%)	16 (14%)	6 (5%)	
	Semester 5–6	9 (13%)	27 (40%)	17 (25%)	12 (18%)	3 (4%)	0.435^1^
	Semester 2–3	9 (20%)	15 (34%)	14 (32%)	4 (9%)	2 (5%)	
Witholding voice (Cronbach ‘s alpha = 0.84, mean IIC = 0.58)							
…did you choose not to bring up your specific concerns about patient safety?	Total	34 (29%)	45 (38%)	25 (21%)	11 (9%)	2 (2%)	
	Semester 5–6	14 (20%)	32 (46%)	14 (20%)	7 (10%)	2 (3%)	0.082^1^
	Semester 2–3	18 (41%)	13 (30%)	10 (23%)	3 (7%)	0 (0%)	
…did you keep ideas for improving patient safety in your unit to yourself?	Total	31 (26%)	37 (32%)	36 (31%)	12 (10%)	1 (1%)	
	Semester 5–6	17 (25%)	19 (28%)	25 (36%)	8 (12%)	0 (0%)	0.13^1^
	Semester 2–3	13 (30%)	18 (41%)	10 (23%)	2 (5%)	1 (2%)	
…did you remain silent when you had information that might have prevented a safety incident in your unit?	Total	67 (57%)	32 (27%)	10 (9%)	5 (4%)	3 (3%)	
	Semester 5–6	36 (52%)	20 (29%)	6 (9%)	5 (7%)	2 (3%)	0.07^1^
	Semester 2–3	30 (68%)	10 (23%)	3 (7%)	0 (0%)	1 (2%)	
…did you not address a colleague (doctors and/or nurses) if he/she didn’t follow important patient safety rules, intentionally or unintentionally?	Total	35 (30%)	39 (33%)	25 (21%)	11 (9%)	7 (6%)	
	Semester 5–6	19 (28%)	22 (32%)	18 (26%)	6 (9%)	4 (6%)	0.231^1^
	Semester 2–3	15 (34%)	17 (39%)	7 (16%)	3 (7%)	2 (5%)	
Speaking up (Cronbach ‘s alpha = 0.77, mean IIC = 0.45)							
…did you bring up specific concerns about patient safety?	Total	28 (24%)	55 (47%)	23 (19%)	10 (8%)	2 (2%)	
	Semester 5–6	18 (26%)	32 (46%)	12 (17%)	7 (10%)	1 (1%)	0.617^1^
	Semester 2–3	9 (20%)	21 (48%)	10 (23%)	3 (7%)	1 (2%)	
…did you address an error which–if uncaptured–could be harmful for patients?	Total	32 (27%)	42 (36%)	27 (23%)	13 (11%)	4 (3%)	
	Semester 5–6	20 (29%)	24 (34%)	18 (26%)	6 (9%)	2 (3%)	0.595^1^
	Semester 2–3	10 (23%)	18 (41%)	8 (18%)	6 (14%)	2 (5%)	
…did you address a colleague (doctors and/or nurses) when he/she didn’t follow important patient safety rules, intentionally or unintentionally?	Total	30 (26%)	53 (45%)	23 (20%)	10 (9%)	1 (1%)	
	Semester 5–6	22 (32%)	27 (39%)	16 (23%)	3 (4%)	1 (1%)	0.093^1^
	Semester 2–3	5 (11%)	26 (59%)	6 (14%)	7 (16%)	0 (0%)	
…did you prevent an incident from occurring as a consequence of bringing up specific concerns about patient safety?	Total	53 (46%)	44 (38%)	11 (9%)	8 (7%)	0 (0%)	
	Semester 5–6	38 (55%)	23 (33%)	6 (9%)	2 (3%)	0 (0%)	0.013^1^
	Semester 2–3	14 (33%)	19 (44%)	5 (12%)	5 (12%)	0 (0%)	

^1^Wilcoxon rank-sum test for independent samples; *IIC* Inter-item correlation

### Speaking up related climate scales

The psychological safety for speaking up significantly differed between semesters and was higher for younger students (*p* = 0.003). Concerning respondents’ perception of an encouraging environment, the mean score was rather low with no significant differences between semesters (*p* = 0.072). There was a higher perceived resignation towards speaking up for older than younger students (*p* = 0.003). For detailed results, see Table 3.

**Table 3:** Means and standard deviations (SD) of speaking up related climate scales

Items organized in scales	Semester 2–3 (*N* = 44)		Semester 5–6 (*N* = 70)		
	M(SD)	Missing values	M(SD)	Missing values	*p* value
**Psychological Safety for Speaking up (Cronbachs alpha = 0.86, mean IIC = 0.55)**	5.3 (1.2)	0	4.5 (1.4)	0	0.003^1^
I can rely on my colleagues (physicians and/or nurses), whenever I encounter difficulties in my work	5.8 (1.4)	0	4.9 (1.6)	0	< 0.001^1^
I can rely on the shift supervisor (person in charge of a shift) whenever I encounter difficulties in my work	5.8 (1.5)	1	4.6 (1.8)	0	< 0.001^1^
The culture in my unit/clinical area makes it easy to speak up about patient safety concerns	4.7 (1.8)	0	4.2 (1.9)	0	0.237^1^
My shift supervisors (person in charge of a shift) react appropriately, when I speak up about my patient safety concerns	5.2 (1.4)	0	4.6 (1.7)	4	0.110^1^
When I have concerns regarding patient safety, it is difficult to submit them	4.8 (1.5)	0	4.1 (1.5)	2	0.018^1^
**Encouraging Environment for Speaking up (Cronbach’s alpha = 0.78, mean IIC = 0.53)**	4.3 (1.7)	0	3.7 (1.7)	1^b^	0.072^1^
In my unit/clinical area, I observe others speaking up about their patient safety concerns	4.2 (1.8)	0	3.6 (1.9)	2	0.052^1^
I am encouraged by my colleagues (physicians and/or nurses) to speak up about patient safety concerns	4.3 (2.3)	0	3.7 (2.2)	2	0.176^1^
I am encouraged by my shift supervisor (person in charge during a shift) to speak up about patient safety concerns	4.4 (2.1)	0	3.7 (2.2)	0	0.088^1^
**Resignation towards Speaking up**^c^ **(Cronbach’s alpha = 0.48, mean IIC = 0.23)**	3.0 (1.2)	0	3.7 (1.2)	2^b^	0.003^1^
Having to remind staff of the same safety rules again and again is frustrating^a^	3.0 (1.9)	0	3.5 (1.7)	5	0.091^1^
Sometimes I become discouraged because nothing changes after expressing my patient safety concerns^a^	3.1 (1.6)	1	4.1 (1.6)	3	0.001^1^
When I have concerns regarding patient safety, it is difficult to submit them^a^	3.0 (1.6)	0	3.6 (1.9)	1	0.139^1^
**Total scale score (Cronbach’s alpha = 0.85, mean IIC = 0.34)**	4.9 (1.0)	0	4.2 (1.2)	0	0.003^1^

^a^Negatively worded items recoded for the total scale score and total scale reliability

^b^Subjects with more missing responses than half of the items in the scale were not considered for the computation of total scale scores; *ICC* Inter-item correlation

^c^This subscale has a low reliability due to the presence of item “When I have concerns regarding patient safety, it is difficult to submit them”, which has a very low correlation with the total scale score (r= 0.16) as compared to the other two items included in this subscale (*r*= 0.35 and *r*= 0.39, respectively). When this item is dropped, Cronbach alpha increases to 0.59

^1^Wilcoxon rank-sum test for independent samples

### Hypothetical situation (vignette) and barriers

**Fig. 1:**
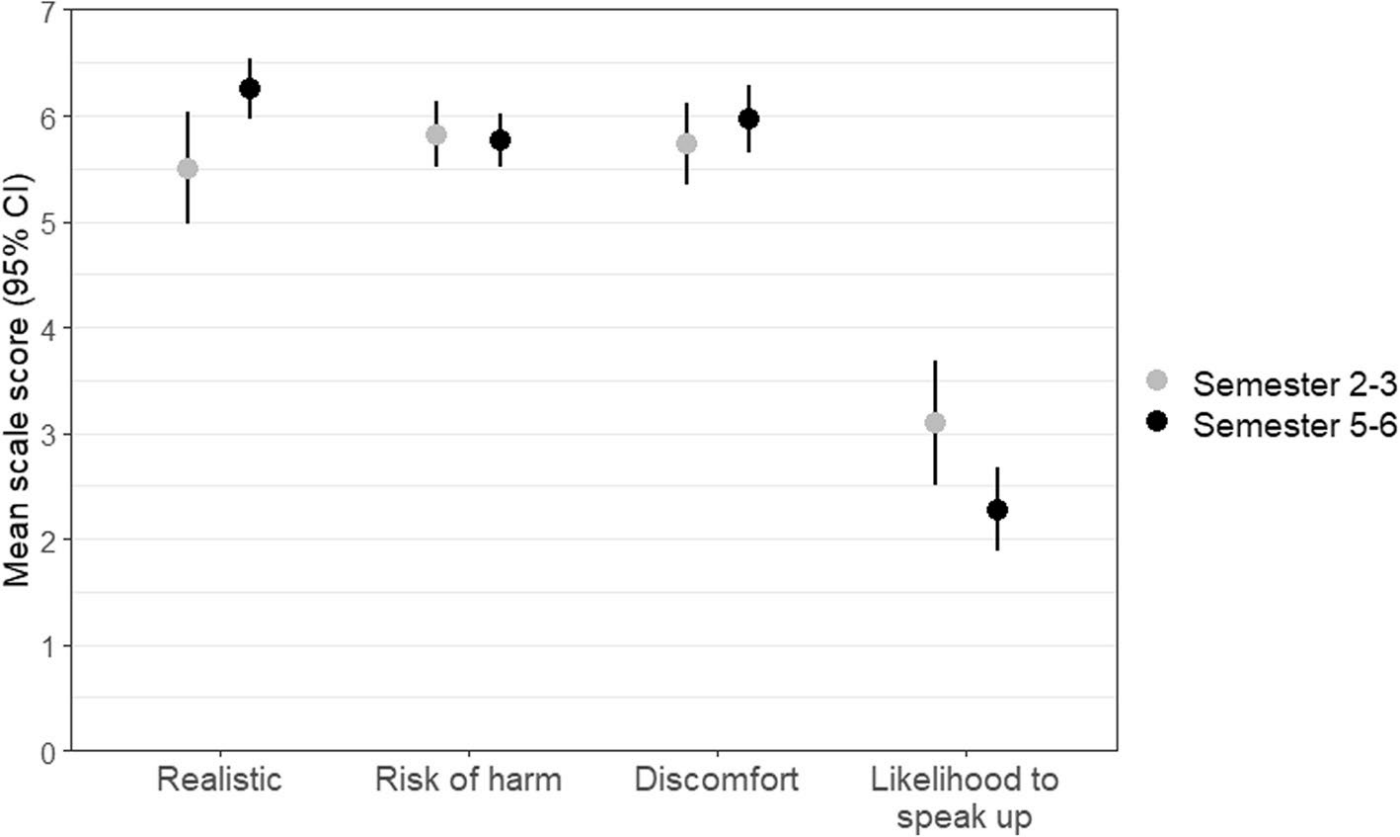
Mean ratings and 95% CI of the missed hand disinfection vignette by study term

The hypothetical situation was rated as a very realistic scenario in a clinical setting, with significantly higher scores among older students (*p* = 0.016). It was also rated as a very dangerous situation. However, the reported likelihood make the consultant aware of the missed hand disinfection was rather low and significantly lower for older students (*p* = 0.027). Nursing students reported they would feel very uncomfortable to instruct the consultant to clean their hands or to wear gloves (see Fig. 1 and Table 4).

**Table 4:** Means and standard deviations (SD) for the hypothetical situation (vignette) for the total, and stratified by study semesters

	All (*N* = 118)	Semester 2–3 (*N* = 44)	Semester 5–6 (*N* = 70)	
	M(SD)	M(SD)	M(SD)	*p* value
How realistic is this situation?	6.0 (1.5)	5.5 (1.7)	6.3 (1.2)	0.016^1^
Risk of harm	5.8 (1.0)	5.8 (1.0)	5.8 (1.0)	0.770^1^
Likelihood to speak up	2.6 (1.8)	3.1 (2.0)	2.3 (1.7)	0.027^1^
Discomfort	5.8 (1.4)	5.7 (1.3)	6.0 (1.3)	0.242^1^

^1^Wilcoxon rank-sum test for independent samples

**Fig. 2:**
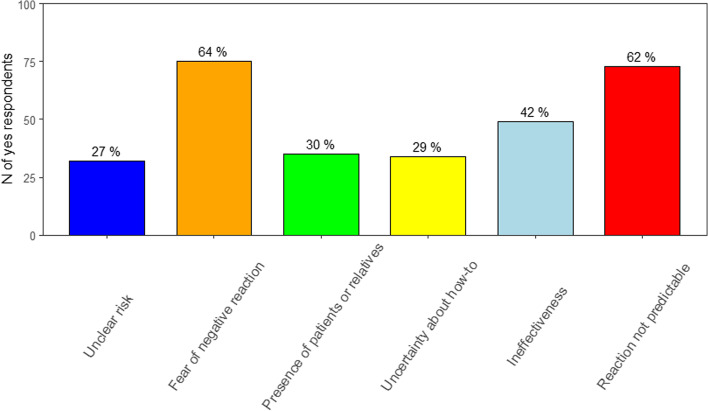
Frequency of relevant barriers for speaking up about Patient Safety concerns

Most relevant barriers to speak up about patient safety concerns were fear of negative reactions (75/118 yes responses, 64%), reaction not predictable (73/118 yes responses, 62%) and ineffectiveness (49/118 yes responses, 42%) (see Fig. 2).

## Discussion

Speaking-up behaviours give a valuable insight into teamwork, safety competencies, culture as well as communication style in a healthcare setting. This study was the first to our knowledge to analyse speaking up behaviours, climate and barriers in nursing students who completed their internship in an Austrian academic center.

Overall, perceived psychological safety was rated good, though the encouraging environment was rated as rather low and resignation towards speaking up was high. The hypothetical vignette was rated as very realistic and dangerous, but respondents reported a low likelihood to report the given situation. The most often noted barrier to speaking up was fear of negative or unpredictable reactions. Most notably, we observed significant group differences between study terms in the items related to speaking-up climate. Most striking was the difference in risk for patient harm ratings among nursing students, a phenomenon that was also observed in a previous study for medical students [6]. Students of higher semesters reported a less favourable speaking up climate compared to their counterparts in earlier semesters. Ito et al. demonstrated that psychological safety is a multilevel phenomenon related to a unit culture that facilitates interpersonal risk behaviour [17]. The unit culture influences proactive behaviours such as asking questions, reporting errors and communicating openly. It further depends on strong interpersonal relationship and an effective culture that includes collaboration and trust. Considering the last two issues, this might strongly influences students behaviours concerning speaking up throughout their internship.

Dinius et al. reported that professional teamwork, safety competencies and communication is a necessity in daily routine which is also influencing patient safety [8, 9, 18]. Furthermore, teamwork and communication have an impact on problem-based and reflective learning and consequently, on critical thinking or problem-solving skills [17, 19]. Lower levels of collegial support increase the likelihood of errors which has a negative impact on patient safety [20].

The safety climate is connected to healthcare workers willingness to speak up [6]. Therefore, speaking-up behaviour also provides an insight into patient safety education and the transformation into clinical practice [11]. In this study, nursing students reported that they noticed rule violations but that they did not speak up in the majority of the cases. On the one hand, evidence exists that patient safety education should be taught throughout the entire curriculum to teach students in order to prevent mistakes in clinical routine and improve safety [21]. On the other hand, a hierarchical system, which was already identified in previous studies is a common barrier for students to transform knowledge into practice [14]. Evidence exists that bullying in the workplace remains an issue [20]. The key to success is the importance of respectful interpersonal relationship between health professionals, mentorship between senior nurses and students or graduates [20].

For nurses, attrition rates of up to 70% are reported, therefore strengthening psychological safety through organizational as well as individual factors seem to be important issues to foster the relationship between an organization and its employees [22]. Furthermore, Song et al. suggested that nurses manifest poor critical thinking attitudes [23], however, these skills are essential to maintain patient safety [23–25].

Results of this study also suggest that nursing students would like to share their critical thoughts as the majority of nursing students had specific concerns. They also reported a rather extensive speaking-up behaviour, but they kept their ideas to themselves. These results could imply that nursing students gave a socially desired answer to the questionnaire. Another reason might be that there are frequent occurrences of trigger situations in combination with sharp trade-offs, in which type of situations raising concerns is acceptable and promising and in which it is not [6].

The transition phase from being a student to a certified nurse is very important to develop self-efficacy, professional skills and thereby job satisfaction [22].

Nursing students are in general attentive observers, however, due to hierarchical structures they fear to speak up, a tendency that was even higher in older students. This may be an indication that older students, who already completed several internships in the respective teaching hospital and other health care institutions, experienced unpleasant situations. Hierarchical academic systems are in general not supportive of reporting errors or rule violations [6]. To conclude, it is important to foster a safety climate in clinical departments to improve patient safety [26]. One possible trigger could be that students education profits through medical simulation as it was reported that intrinsic motivation of students significantly increase after simulation based training [27].

## Limitations

Although the response rate was rather high, the study population only consisted of 118 nursing students. Very few of them attended semesters 2 and 6. No data are available from non-responders and results may be subject to self-selection bias. The sample size most likely affected reliability scores. Resignation towards speaking up indeed showed a low Cronbach alpha of 0.48. In the study by Schwappach et al. [14] this scale had the lowest reliability (0.66), though higher than in the current study most likely due the larger sample size (*n* = 118 versus *n* = 326). The low reliability of this subscale suggests a somewhat inconsistent response behaviour among students. The results of this scale should thus be interpreted with caution and its items should be revised in a future version of the questionnaire.

Students of the study term 2017, who were asked to participate in the study in the year 2020, were invited using an online-survey instrument (Evasys©) due to COVID-19. The response rate for the online group was very low compared to the paper-based survey. One likely reason for this difference in the response rate might be the context of the survey administration. While nursing students using the paper-and-pencil questionnaires were invited during a lesson, and therefore all students were aware of the invitation to complete the survey, the online survey was simply sent to students per e-mail and might have received less attention. We cannot exclude that some e-mails ended up in the spam folders and were never read. In this sense, the survey method had an influence on the response behaviour. Furthermore, generalizability of results is limited as the study was performed in one single institution.

## Conclusions

Identifying speaking-up behaviours is vital to determine risk behaviours. This study showed that nursing students are equipped with patient safety competencies during their education; however, hierarchical barriers in the clinical setting do not encourage their utilization. Therefore, effective training which can influence organizational and individual development is needed [18, 28]. Furthermore, the current study and others suggest that all healthcare professionals should be included in efforts to improve teamwork and communication skills [9]. Creating a supportive environment in terms of patient safety needs teams that improve learning, communication and performance within organizations [29]. It is recommended to assess students’ self-perceived competencies during and after their education or internships which might help to adjust curricula to student´s needs [9].

## Supplementary Information

**Additional file 1.**

## Data Availability

The datasets used and/or analysed during the current study are available from the corresponding author on reasonable request.

## Abbreviations

SUPS-Q: Speaking Up about Patient Safety Questionnaire
Covid-19: Coronavirus disease 2019
